# Supplementary material for: Phylogenetic context of antibiotic resistance provides insights into the dynamics of resistance emergence and spread
Source: medRxiv. 2025 Jun 5:2025.06.04.25328982. Preprint. [Version 1] doi: 10.1101/2025.06.04.25328982 (PMC12154989; doi:10.1101/2025.06.04.25328982)
Supplement: Supplement 1 [file NIHPP2025.06.04.25328982v1-supplement-1.pdf]

SUPPLEMENTAL MATERIAL

Supplementary Table 1. Minimum-inhibitory concentration breakpoints applied in this study

Agent	Susceptible	Resistance	
		Intermediate	Resistant
Trimethoprim-sulfamethoxazole[1]	≤ 8/40 µg/mL	-	≥16/80 µg/mL
Gentamicin	≤ 4 µg/mL	8 µg/mL	≥ 16µg/mL
Amikacin	≤ 4 µg/mL	8 µg/mL	≥ 16µg/mL
Colistin	-	≤ 2µg/mL	≥ 4µg/mL
Imipenem-relebactam	≤ 1/4 µg/mL	2/4 µg/mL	≥ 4/4 µg/mL
Meropenem-vaborbactam	≤ 4/8 µg/mL	8/8 µg/mL	≥ 16/8 µg/mL

[1.] Due to abstraction from the regional clinical microbiology's reporting practice (sulfamethoxazole doubling from 20 µg/mL to 320 µg/mL, we adjusted the 2024 Clinical and Laboratory Standards Institute breakpoints for trimethoprim-sulfamethoxazole accordingly. All remaining antibiotics used susceptibility testing breakpoints from the 2021 Clinical and Laboratory Standards Institute's Performance Standards for Antimicrobial Susceptibility Testing report.

Supplemental Table 2. Variables considered for multivariable logistic regression modeling

Variables	Type	Description
**Demographics**		
Age	Continuous	Age in years
Sex	Binary	Female or male sex
Length of stay	Continuous	Days in the facility preceding isolate collection
**Indwelling devices**	Binary	Presence of the device on the day of isolate collection
Tracheostomy tube		
Central venous catheter		
Indwelling urinary catheter		
Gastrostomy tube		
**Comorbidities**	Binary	Presence of comorbidities on the day of isolate collection
Acute kidney injury		
Chronic kidney disease		
Ventilator-dependent respiratory failure		
Underweight or malnourished		Body mass index $\leq$ 18.5 kg/m^2
Congestive heart failure		
Decubitus ulcer stage IV/V		
COPD or chronic bronchitis		Chronic obstructive pulmonary disease or chronic bronchitis
Brain injury		Anoxic brain injury from a cerebral vascular accident, causing paresis.
Malignancy		
Obesity		Body mass index $\geq$ 30 kg/m^2
**Antibiotic exposures in the preceding 30 days**	Binary	Exposure of $\geq$1 day of antibiotics modeled as a binary variable
Intravenous vancomycin		
Carbapenem		Exposure to ertapenem, imipenem, and/or meropenem
Cephalosporin		Exposures to third-, fourth-, and/or fifth-generation cephalosporins, notably ceftriaxone, ceftazidime, cefepime, or ceftaroline
Aminoglycoside		Exposures to amikacin, tobramycin, and/or gentamicin. Aminoglycoside exposure was modeled separately, as gentamicin and amikacin resistance had distinct distributions across the phylogenetic tree
Metronidazole		
Fluoroquinolone		Exposure to levofloxacin and/or ciprofloxacin
Linezolid		

Tigecycline		
Piperacillin-tazobactam		
Polymyxin		Exposure to polymyxin B and/or colistin
Daptomycin		
Aztreonam		
Trimethoprim-sulfamethoxazole		

Supplemental Table 3. Characteristics of the study population at the time of first isolate collection

Characteristic	Overall (n=312)
**General characteristics**	
Age, y, median (IQR)	72.72 (18.2)
Female Sex, no. (%)	149 (47.8)
Length of stay before culture, median (IQR)	21 (37)
**Indwelling device presence**	
Device count, median (IQR)	2 (2)
Any device present	274 (87.8)
Tracheostomy tube	219 (70.2)
Central venous catheter	180 (57.7)
Indwelling urinary catheter	174 (55.8)
Gastronomy Tube	117 (37.5)
**Medical comorbidities**	
Comorbidity count, median (IQR)	2 (3)
Presence of multiple comorbidities	201 (64.4)
Acute kidney injury	152 (48.7)
Chronic kidney disease	107 (34.3)
Ventilator-dependent respiratory failure	96 (30.8)
Underweight or malnourished	82 (26.3)
Congestive heart failure	65 (20.8)
Decubitus ulcer stage IV/V	65 (20.8)
COPD or chronic bronchitis	61 (19.6)
Brain injury	59 (18.9)
Malignancy	39 (12.5)
Obesity	17 (5.4)
**Systemic antibiotic exposure in the preceding 30 days**	

Any antibiotic exposure	227 (72.8)
Intravenous vancomycin	138 (44.2)
Carbapenem	99 (31.7)
Cephalosporin	79 (25.3)
Aminoglycoside	63 (20.2)
Amikacin	40 (12.8)
Tobramycin	21 (6.7)
Gentamicin	11 (3.5)
Metronidazole	60 (19.2)
Fluoroquinolone	58 (18.6)
Linezolid	46 (14.7)
Tigecycline	42 (13.5)
Piperacillin-tazobactam	42 (13.5)
Polymyxin	37 (11.9)
Daptomycin	12 (3.8)
Trimethoprim-sulfamethoxazole	11 (3.5)
Aztreonam	10 (3.2)

Supplemental Table 4. Comparison of ancestral state reconstruction models using Akaike information criteria

Phenotype	Model	No. parameters	Rate 1[1]	Rate 2	Log likelihood	AICc	Best model[2]
TMP-SMX	ER	1	12301.25	NA	-186.86	375.72	ARD
TMP-SMX	ARD	2	14266.6	9772.79	-185.73	375.49	
Gentamicin	ER	1	10271.29	NA	-162.08	326.17	ER
Gentamicin	ARD	2	10791.82	9820.55	-162.01	328.05	
AMK	ER	1	23413.38	NA	-226.98	455.96	ARD
AMK	ARD	2	24663.74	42368.73	-222.47	448.97	
Colistin	ER	1	8707.65	NA	-159.26	320.53	ER
Colistin	ARD	2	7875.24	11609.37	-158.5	321.02	
BL/BLI	ER	1	7843.71	NA	-166.53	335.06	ARD
BL/BLI	ARD	2	12924.48	76998.04	-145.38	294.79	

Abbreviations: AICc, sample-size corrected Akaike information criterion; ARD, all rates different; BL/BLI, beta-lactam/beta-lactamase inhibitor; ER, equal rates; TMP-SMX, trimethoprim-sulfamethoxazole

[1]. Under the all rates different model, rate 1 represents transitions from susceptible to resistant, while rate 2 represents transitions from resistant to susceptible.

[2]. The model with the lowest Akaike information criterion value for each phenotype was chosen for downstream analyses.

Supplemental Table 5. Differences in phylogenetic clustering of antibiotic resistance across clades of *Klebsiella pneumoniae* sequence type 258

Phenotype	Clade	Resistant isolates	Resistance frequency	Phylogenetic events	Singletons	Singleton isolates	Clusters	Cluster isolates	Cluster size median (range)	Phylogenetic frequency	Clustering frequency
TMP-SMX	I	40	29.85%	19	14	18	5	22	4 (2-8)	16.81%	26.32%
TMP-SMX	II	167	66.27%	18	14	14	4	153	2.5 (2-146)	9.33%	22.22%
Gentamicin	I	36	26.87%	18	13	17	5	19	3 (2-7)	11.84%	27.78%
Gentamicin	II	163	64.68%	9	6	6	3	157	8 (2-147)	6.29%	33.33%
Amikacin	I	79	58.96%	21	13	14	8	65	7 (2-25)	7.34%	38.10%
Amikacin	II	53	21.03%	26	21	24	5	29	4 (2-15)	8.58%	19.23%
Colistin	I	19	14.18%	14	13	13	1	6	6 (6-6)	8.92%	7.14%
Colistin	II	110	43.65%	14	7	7	7	103	4 (2-71)	5.83%	50.00%
BL/BLI	I	37	27.61%	21	16	17	5	20	2 (2-10)	15.11%	23.81%
BL/BLI	II	19	7.54%	17	16	17	1	2	2 (2-2)	6.80%	5.88%

Abbreviations: BL/BLI, beta-lactam/beta-lactamase inhibitor; TMP-SMX, Trimethoprim-Sulfamethoxazole.

[1.] Phylogenetic frequency accounts for the number of episodes a trait occurs across the phylogenetic tree relative to the total number of possible events.

[2.] Clustering frequency characterizes the proportion of phylogenetic events that are phylogenetic clusters.

Supplemental Table 6. Evaluation of single-regime and clade-specific evolutionary rate model using *fitmultiMK*

		***fitmultiMK* single-regime model**				***fitmultiMK* two-regime model**					**Model comparison via the likelihood ratio test**		
**Phenotype**	**Model**	**Rate 1**	**Rate 2**	**Log Likelihood**	**AIC**	**Clade I Rate 1**	**Clade I Rate 2**	**Clade II Rate 1**	**Clade II Rate 2**	**Log Likelihood**	**AIC**	**X2 value**	**p-value**
TMP-SMX	ER	12300.44	NA	-186.86	375.71	12935.96	NA	12016.96	NA	-186.84	377.67	0.04	0.843
TMP-SMX	ARD	14277.61	9773.99	-185.75	375.49	13558.78	18657.04	18620.48	9281	-184.26	376.51	2.98	0.225
Gentamicin	ER	10271.16	NA	-162.08	326.16	15179.17	NA	8494.82	NA	-160.89	325.77	2.39	0.122
Gentamicin	ARD	10765.84	9852.66	-162.02	328.04	18208.26	41250.16	9512.24	8328.1	-158.65	325.29	6.75	0.034
Amikacin	ER	23429.23	NA	-226.98	455.95	398075.94	NA	12357.89	NA	-212	428	29.95	<0.001
Amikacin	ARD	24665.84	42370.76	-222.47	448.94	202426.53	140691.98	16090.46	55069.54	-202.83	413.67	39.27	<0.001
Colistin	ER	8707.39	NA	-159.26	320.52	9924.87	NA	8150.87	NA	-159.14	322.27	0.25	0.616
Colistin	ARD	7889.8	11658.15	-158.52	321.04	184614.13	1097808.65	8487.38	7764.6	-146.18	300.36	24.68	<0.001
BL/BLI	ER	7844.06	NA	-166.53	335.05	22055.86	NA	4480.41	NA	-158.75	321.51	15.54	<0.001
BL/BLI	ARD	12926.43	77010.14	-145.38	294.75	45899.85	139580.52	8497.99	82031.48	-142.39	292.77	5.98	0.05

Abbreviations: AIC, Akaike information criterion; ARD, all rates different; BL/BLI, beta-lactam/beta-lactamase inhibitor; ER, equal rates; TMP-SMX, Trimethoprim-Sulfamethoxazole.

Supplemental Table 7. Modeling of single- and multi-rate categories using *corHMM*

Phenotype	Model	Rate 1	Rate 2	Single-rate Log Likelihood	Single-rate AICc	Category I Rate 1	Category I Rate 2	Category II Rate 1	Category II Rate 2	Multi-rate Log Likelihood	Multi-rate AICc	Best model
TMP-SMX	ER	12301.83	NA	-186.86	375.72	1.44e+12	NA	8.14e-07	NA	-162.37	332.84	Two rate categories
TMP-SMX	ARD	14265.06	9773.29	-185.73	375.49	3.15e+11	3.90e+11	3251.3	1.91e-06	-160.25	332.73	Two rate categories
Gentamicin	ER	10270.57	NA	-162.08	326.17	6.30e+11	NA	0	NA	-157.21	322.52	Two rate categories
Gentamicin	ARD	10792.83	9821.52	-162.01	328.05	3.60e+11	1.18e+12	0.01	4660.81	-156.67	325.55	Two rate categories
Amikacin	ER	23430.82	NA	-226.98	455.96	5.96e+05	NA	1782.15	NA	-202.12	412.35	Two rate categories
Amikacin	ARD	24662.95	42366.02	-222.47	448.97	4.84e+05	7.47e+05	4859.19	51051.42	-197.35	406.92	Two rate categories
Colistin	ER	8707.02	NA	-159.26	320.53	8.61e+17	NA	1326.06	NA	-152.17	312.45	Two rate categories
Colistin	ARD	7876.33	11610.01	-158.5	321.02	1.71e+06	1.31e+05	43726.54	293.61	-147.27	306.76	Two rate categories
BL/BLI	ER	7844.23	NA	-166.53	335.06	1.96e+17	NA	0	NA	-134.75	277.6	Two rate categories
BL/BLI	ARD	12924.8	76995.92	-145.38	294.79	1.35e+06	4.66e+05	1.02e-09	1903.25	-129.65	271.53	Two rate categories

Abbreviations: AICc, sample-size corrected Akaike information criterion; ARD, all rates different; BL/BLI, beta-lactam/beta-lactamase inhibitor; ER, equal rates; TMP-SMX, Trimethoprim-Sulfamethoxazole.

Supplemental Figure 1. Minimum-inhibitory concentration histogram for each antibiotic of interest

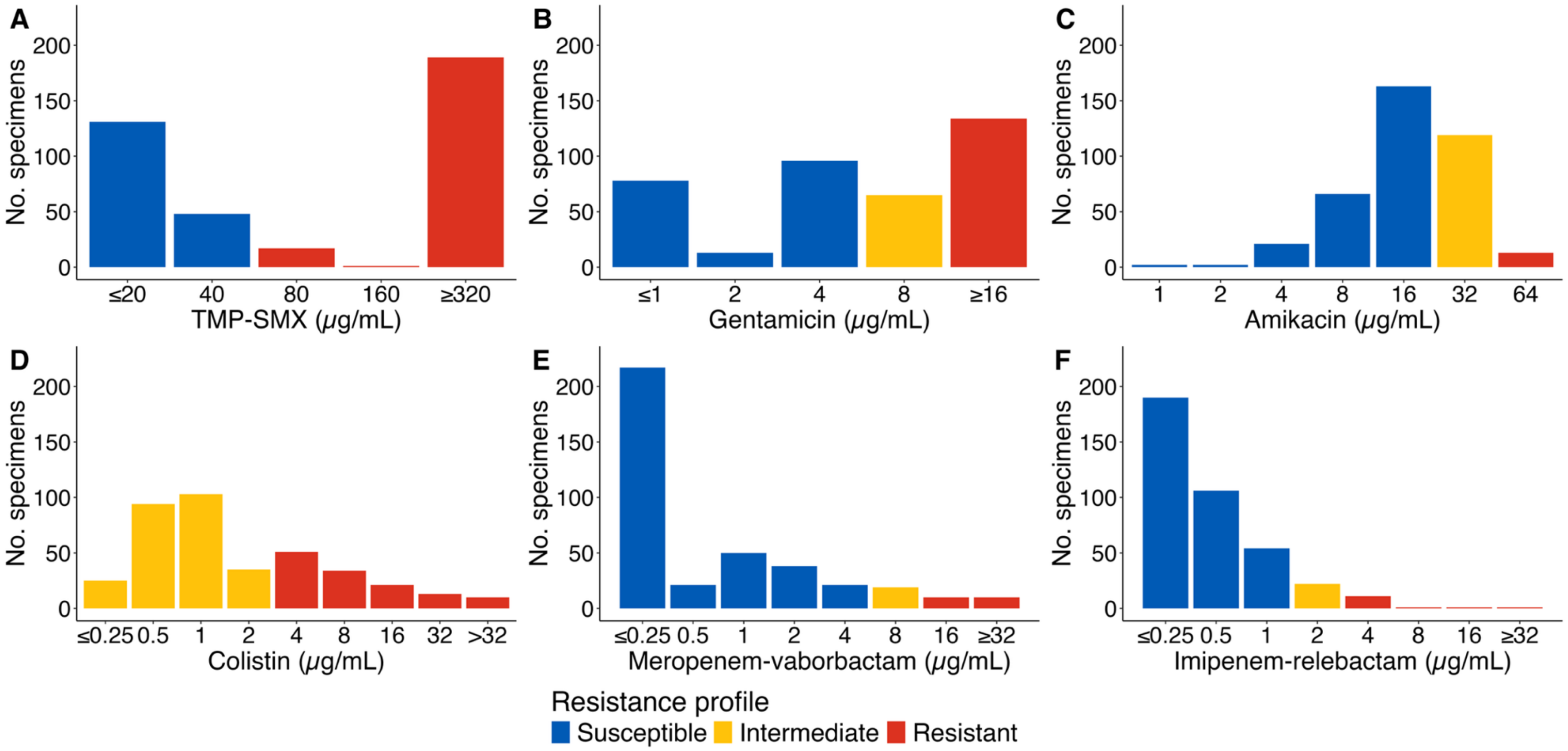

Abbreviations: TMP-SMX, trimethoprim-sulfamethoxazole

Supplemental Figure 2. Ancestral state reconstruction and evolutionary history inferences on the phylogenetic tree

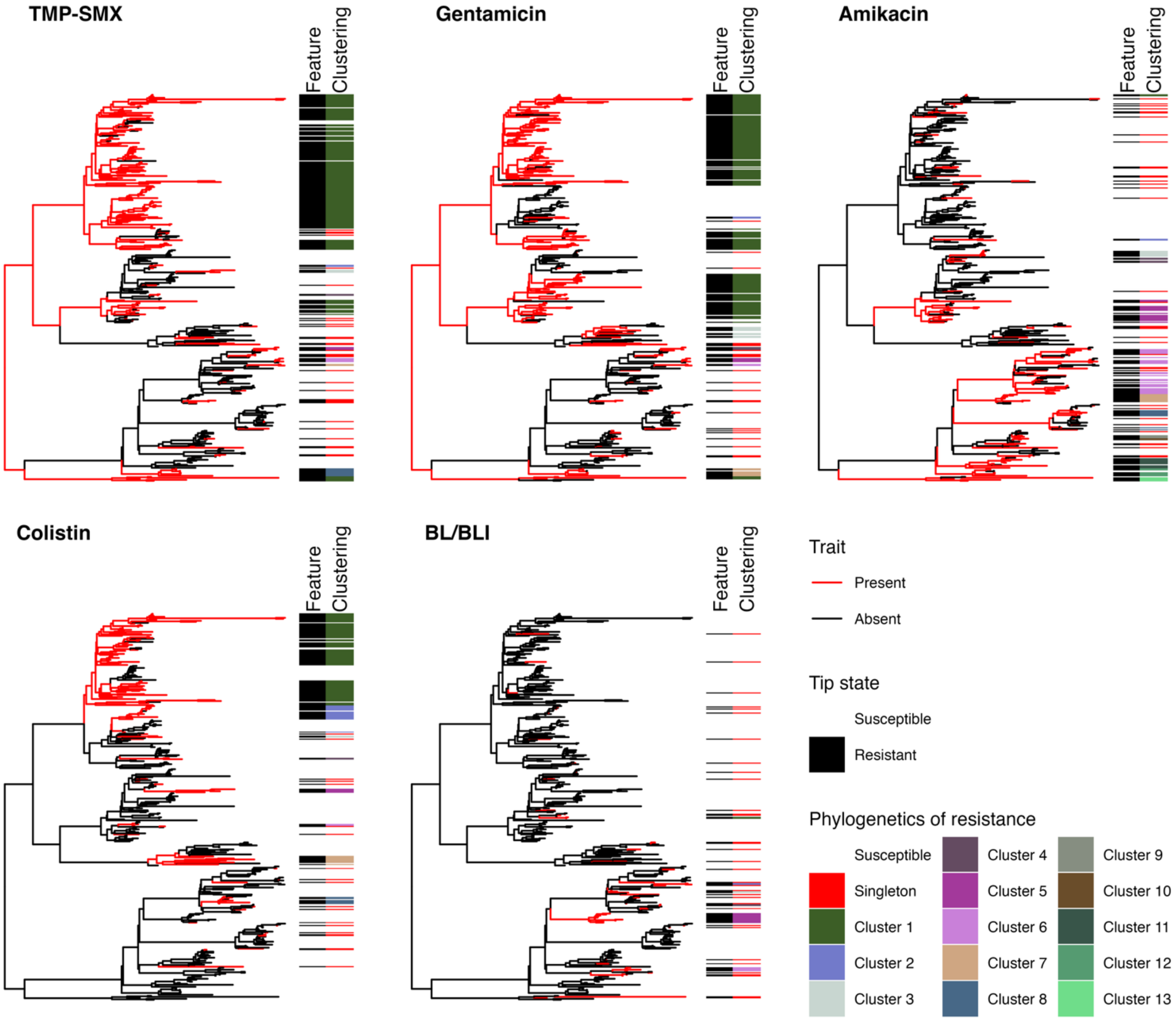

Abbreviations: BL/BLI, beta-lactam/beta-lactamase inhibitors; TMP-SMX, trimethoprim-sulfamethoxazole
